# Incursion of H5N8 high pathogenicity avian influenza virus (HPAIV) into gamebirds in England

**DOI:** 10.1017/S0950268821002740

**Published:** 2022-02-10

**Authors:** Sharon M. Brookes, Karen L Mansfield, Scott M. Reid, Vivien Coward, Caroline Warren, James Seekings, Tanis Brough, Davina Gray, Alejandro Núñez, Ian H. Brown

**Affiliations:** 1 Animal and Plant Health Agency (APHA), New Haw, Surrey, UK; 2 Animal and Plant Health Agency (APHA), Merrythought, Calthwaite, Penrith, Cumbria, UK; 3 Animal and Plant Health Agency (APHA), West House, Thirsk, North Yorkshire, UK

**Keywords:** avian, gamebirds, H5N8, influenza, pheasants

## Abstract

The 2016–17 European outbreak of H5N8 HPAIV (Clade 2.3.4.4b) affected a wider range of avian species than the previous H5N8 outbreak (2014–15), including an incursion of H5N8 HPAIV into gamebirds in England. Natural infection of captive-reared pheasants (*Phasianus colchicus*) led to variable disease presentation; clinical signs included ruffled feathers, reluctance to move, bright green faeces, and/or sudden mortality. Several birds exhibited neurological signs (nystagmus, torticollis, ataxia). Birds exhibiting even mild clinical signs maintained substantial levels of virus replication and shedding, with preferential shedding via the oropharyngeal route. Gross pathology was consistent with HPAIV, in gallinaceous species but diphtheroid plaques in oropharyngeal mucosa associated with necrotising stomatitis were novel but consistent findings. However, minimal or modest microscopic pathological lesions were detected despite the systemic dissemination of the virus. Serology results indicated differences in the timeframe of exposure for each case (*n* = 3). This supported epidemiological conclusions confirming that the movement of birds between sites and other standard husbandry practices with limited hygiene involved in pheasant rearing (including several fomite pathways) contributed to virus spread between premises.

## Introduction

The 2016–17 outbreak of H5N8 (clade 2.3.4.4b) high pathogenicity avian influenza virus (HPAIV) rapidly spread throughout Europe principally via migratory birds, and the virus was detected in a wide range of wild bird species in a number of European countries, including Great Britain (GB). Additionally, the virus caused outbreaks in domestic poultry, in both commercial and small holding settings, leading to clinical signs and mortality in a range of avian species including chickens, turkeys and ducks [1]. Previous studies have shown that the H5N8 strain causing the 2016–17 outbreak was genetically distinct from H5N8 HPAIVs detected during the previous 2014–15 (clade 2.3.4.4a) outbreak, and confirmed the 2016–17 viruses to be novel reassortant strains that had evolved from H5N8 viruses first detected in wild birds in southern Russia and Mongolia in early 2016, but had subsequently acquired new polymerase (PA) and nucleoprotein (NP) gene segments from wild bird progenitors [2]. These genetic changes may explain why the 2016–17 viruses had been more pathogenic in wild birds, compared with H5N8 HPAIV's (clade 2.3.4.4a) isolated during the previous 2014–2015 outbreak [3]. Additionally, the rapid spread throughout Europe and a large number of wild bird detections, suggested that the 2016–17 strain of H5N8 virus had evolved to be more efficiently transmitted between wild and domestic birds [2].

The first detection of the 2016–17 strain of H5N8 HPAIV in GB poultry was confirmed on 16th December 2016 in a turkey flock in England, coinciding with the first wild bird detection in wild Eurasian wigeon (*Anas penelope*) found dead in Carmarthenshire, Wales. There were subsequently a number of wild bird detections (*n* = 25 events) throughout GB in a wide range of species, together with the confirmation of the disease in further domestic poultry settings involving turkeys, chickens and ducks with a total of 13 infected premises. This report describes the incursion of H5N8 HPAIV into the gamebird sector in northern England, involving three linked premises located in the same geographical area.

## Materials and methods

### Clinical background

**Fig. 1.**
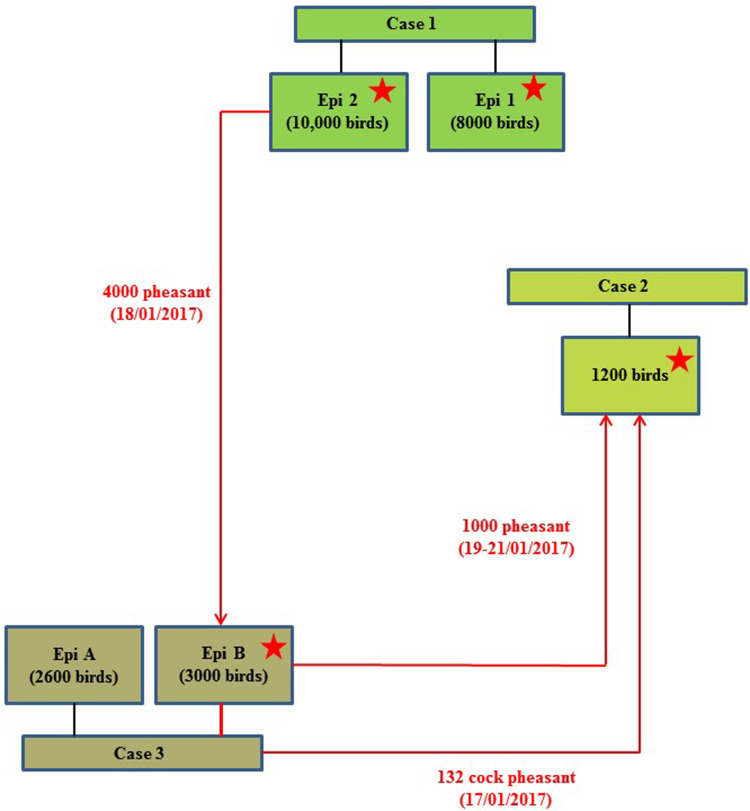
Schematic of the direction of movement between the three infected properties associated with the pheasant cluster. Positive detection of H5N8 viral RNA by RRT-PCR denoted by stars.

On 24th January 2017, following clinical suspicion, H5N8 HPAIV was confirmed in a commercial gamebird farm in England, followed closely by two further infected gamebird premises in the same production pyramid and area on 27th and 30th January 2017. Table 1 summarises the timeline and sampling frames for this pheasant cluster. The three properties were under a common management system/ownership with shared labour, vehicles and equipment. Very limited hygiene measures were in place which is typical for this sector. Documentation upon veterinary enquiry was very limited. Biosecurity measures were also very limited and only basic hygiene practices were in use that led to multiple options for virus spread. There were complex interactions between the three properties, as a result of the processes involved in gamebird breeding and rearing, which are summarised in Figure 1.

**Table 1.** Statutory disease investigation: case submission summary and timeline for each of the pheasant infected properties

	Case 1 (Epi 1 and Epi 2)	Case 2	Case 3* (Epi A and Epi B)
Site background	Originally 18 000 pheasant (two fields; 8000 and 10 000)	Originally 1200 pheasant (in 120 separate netted pens)	2600 and 3000 pheasant in two settings (raised beds and caged)
Date samples collected	23/01/2017	26/01/2017	29/01/2017
Samples submitted per Epi group	20 OP swabs 20 C swabs 20 blood samples Two carcasses	21 OP swabs 21 C swabs 21 blood samples Three carcasses	20 OP swabs (Epi A) 20 C swabs (Epi A) 20 blood samples (Epi A) Four carcasses (Epi A) Three carcasses (Epi B)
Date outbreak confirmed	24/01/2017	27/01/2017	30/01/2017

OP, oropharyngeal; C, cloacal.

* Case 3 underwent initial investigation as a contact premises on 27 January 2017; all swabs and bloods were negative (60/60/60).

#### Case 1

Clinical disease suspicion first arose on 16th January 2017, at a large gamebird property with approximately 18 000 birds (a combination of Blackneck and Polish pheasants *Phasianus colchicus*). Some pheasants had presented with swollen heads and blindness, and the owners initially suspected *Mycoplasma* infection. The pheasants were not housed, but were held in two over-wintering fields (total acreage approximately 60–75 acres), consisting of chicken-wire enclosures without overhead netting (i.e. open topped).

The pheasants had bands on their wings to prevent flying. These two fields were treated as two distinct epidemiological (Epi) groups, Epi group 1 (originally contained approximately 8000 pheasants) and Epi group 2 (originally contained 10 000 pheasants), reared separately but with the same husbandry teams. Suspicion of disease was reported to the UK Competent Authority (Defra) on 23rd January 2017. It was difficult to accurately estimate the percentage mortality, although there had been no obvious clinical signs detected the previous day, many of the birds were not clearly visible across the fields, and in particular the birds in Epi group 1 had been partially quarantined due to the suspicion of *Mycoplasma* infection. Hence it is possible that clinical signs may have inadvertently been missed during inspections. The clinical presentation of the disease was suspected to have been exacerbated by handling stress on 23rd January, when Epi group 2 was being caught prior to intended relocation. Neurological signs and mortality were observed during this catching process. Post mortems of 12 dead birds from each Epi group, carcasses all in apparently good condition, were undertaken by private veterinarians; a consistent finding was lung congestion, although there were no other observations of note. By 24th January 2017, birds in Epi group 1 were exhibiting signs of severe clinical disease, with approximately 60% mortality, and an estimated additional 15% morbidity. Affected birds presented with a hunched appearance, ruffled feathers, closed eyes, reluctance to move and bright green faeces. Several birds were exhibiting neurological signs, including nystagmus, torticollis and ataxia. The remaining 25% of the birds in Epi group 1 appeared clinically normal. In comparison, birds in Epi group 2 exhibited only minimal morbidity; the only observations were reluctance to move and a hunched appearance in some birds. Several dead birds were also observed, but not an excessive number and considered to not constitute excess mortality above baseline numbers. ‘Normal’ mortality rate on this property was between 5 and 10 dead birds per day (in total for both fields).

This property was part of a complex housing system involving multiple sites under common management, leading to the identification of a number of high-risk contact properties.

#### Case 2

A game bird rearing farm with approximately 1200 pheasants held in 120 outdoor netted pens (four rows of thirty pens); each pen housed approximately ten birds (one cock and nine hens). This site had been identified as a ‘contact’ property to Case 1 (Epi group 2), following the delivery of a batch of 1000 pheasant hens originating from Case 1 site (delivered indirectly 19th–21st January 2017, see Case 3 below). It was ascertained that prior to delivery to Case 2 site, these 1000 pheasants had actually been transferred to another property (later confirmed as Case 3) for breeding purposes as part of a larger batch of 4000 birds, for vaccination as described below. Additionally, there had previously been a delivery of cock pheasants to Case 2 site (*n* = 132) from Case 3 site (17th January 2017).

Suspicion of disease (Case 2) was officially reported on 26th January 2017 during the APHA tracing inspection. A small number of pheasants were presenting with clinical signs suggestive of disease (lethargy, neurological signs, tremor, shaking) in 2/120 pens, potentially exacerbated by the stress of handling. There had also been six recent sudden deaths since 22nd January 2017 following the delivery of pheasants from Case 1 property. Post mortems of four birds were undertaken by APHA/Defra veterinarians; small intestinal haemorrhages were detected in two out of four birds, although there were no other observations.

#### Case 3

A game bird farm with approximately 63 000 birds of different breeds and species (pheasants plus a small number of fancy chickens, geese, ducks, and partridge), held within a variety of management groups and penning formats at eleven different settings on the site. The pheasants were held in open fields, raised breeding pens or netted cages, and many were currently housed in breeding groups of approximately ten birds (one cock and nine hens). It was reported that the birds in Case 3 site Epi group B had originated from the site associated with Case 1 (Epi group 2), from where 4000 pheasant hens had been delivered to Case 3 site for vaccination on 18th January 2017. Approximately 1000 of these pheasants had then been transferred to Case 2 site for breeding purposes as described above between 19th and 21st January 2017, whereas 3000 remained at Case 3 site (Epi group B). Therefore the pheasants at this case site had been identified as a contact tracing to the previous case (Case 1), and pheasants within Epi group B underwent initial laboratory investigations as a tracing property on 27th January 2017 (enhanced sampling at 60/60/60 – cloacal (C) swabs, oropharyngeal (OP) swabs, bloods; taken from the population of birds transferred from Case 1) but all laboratory results at this stage were negative. There was a little reported movement of birds between each of the different settings on Case 3 site, although there was husbandry staff and vehicle movement. During enhanced APHA surveillance inspections at Case 3 site on 29th January 2017, mortalities were observed in one setting which contained a total of 2600 pheasants (Epi group A), where birds were housed in raised stainless steel cages (approximately ten birds per cage), with mesh floor and roof, and solid sheet metal sides. Clinical presentation included neurological signs, ataxia and torticollis. Statutory disease investigation sampling was conducted from within Epi group A. Additionally, in a further caged setting (Epi group B) which contained 3000 birds in breeding cages, some mortalities were also observed. Since this was the high-risk group of pheasants which had been traced from Case 1, three carcasses from this group were also submitted for testing. No additional samples were taken from further pheasants groupings within Epi group B.

### Statutory disease investigation on clinical suspicion

In all cases, disease suspicions were reported to the Competent Authority (Defra), official samples were taken in accordance with standard protocols [4] and submitted to APHA for diagnosis as official statutory disease investigations. The premises were placed under restriction according to legislative requirements (EU AI directive 1994/2008). Diagnostic samples consisted of C swabs, OP swabs and clotted blood samples (20/20/20) from each suspect Epi group, along with carcasses if available. In general, sampling frames were applied on a precautionary basis testing for a lower prevalence given the uncertainties around efficient transmission. Randomised selection of cages was done for sampling. The sampling frame was based on 95% confidence of detecting 5% prevalence and followed the guidance set out in the EU AI diagnostic manual [4]. Field epidemiologists on the ground collecting official samples satisfied themselves the cohort for sampling was of the stated origin.

Sampling frames are specified in the competent authority's standard instruction and are based on those set out in the EU AI diagnostic manual.

Due to the transfer of suspected infected birds from one large epi unit (Case 1, Epi group 2) into many smaller breeding units of ten birds, a bespoke enhanced sampling strategy was implemented for these contact traced breeding pheasant units. Three out of ten birds per breeding pen were sampled, from twenty pens across the setting totalling 60C/60OP/60 bloods). This bespoke submission sampling strategy was used at all contact tracing pheasant breeding sites (including Case 3, Epi group B). However, the subsequent official statutory disease investigation for Case 3 involved the submission of the standard set of samples (20/20/20) as described above.

Samples collected from the three cases are summarised in Table 1. Subsequent samples were submitted following cull, or as a result of tracings due to epidemiological linkages for source or spread and/or zonal surveillance for proving disease freedom.

### Molecular detection of viral RNA

RNA was extracted from the original clinical material (single or pooled OP or C swabs, and tissue suspensions prepared from carcasses (if submitted)) using a customised version of the QIAmp viral RNA BioRobot kit in conjunction with a Universal BioRobot (Qiagen, Manchester, UK). Extracted RNA from statutory disease investigation samples were assessed using four AIV real-time reverse transcription-polymerase chain reaction (RRT-PCR) assays: (i) the Matrix (M)-gene assay for generic detection of influenza A virus using the primers and probes of Nagy *et al*. (2010) [5]; (ii) H5 and (iii) H7 AIV RRT-PCR assays to test for notifiable AI (NAI) [6, 7], and (iv) an N8-specific RRT-PCR using primers and probe for detection of 2016-cluster H5N8 viruses, with the chemistry as for the M-gene, H5 and H7 RRT-PCR assays [8]. Extracted RNA from samples taken at cull or for epidemiological investigations into source and spread tracing were not tested using the H7 RRT-PCR. All samples producing a threshold cycle (Ct) value <36.0 by any of these four AIV RRT-PCRs were considered positive [9]. For initial statutory disease investigation samples only, RNA extracts were also screened for the presence of avian paramyxovirus type 1 (APMV-1) to include Newcastle disease (ND) virus, utilising primers and probes targeting the L-gene of APMV-1 [10]. A positive result using this assay was denoted by a Ct value <37.0. All amplifications were carried out in an Mx3005P qPCR System (Agilent).

### Pathology and immunohistochemistry

Post mortem examinations of the carcasses submitted for testing were carried out and tissues collected. In each Epi unit, four standard tissue pools were collected from the submitted birds for molecular testing and virus isolation (numbers of birds detailed in Table 2): brain, lung and trachea, intestine (caecal tonsil and jejunum) and mixed viscera (heart, kidney, spleen and liver). Samples from individual birds were also fixed in neutral buffered formalin for a minimum period of five days for histopathological examination and detection of influenza A nucleoprotein by immunohistochemistry (IHC) using standard methods [11].

**Table 2.** Laboratory analyses: summary of results at bird level for serology (detection of haemagglutination inhibition antibodies by HAIT test) and swabs (detection of viral RNA by H5 RRT-PCR)

Case	Epi Group	Date of sampling	Time of sample	No. tested	Serology				PCR
					% Neg	% Inc/IS	% Non-Neg	% Pos	% Pos
**1**	**1**	24/01/17	Disease Suspicion	20	30 (6/20)	10 (2/20)	35^a^ (7/20)	25^b^ (5/20)	100
	**2**	24/01/17	Disease Suspicion	20	100	0	0	0	100
	**1&2**	24/01/17	Carcasses	1 + 1	*Na*	*na*	*Na*	*na*	100
	**1**	27/01/17	Cull	15	73.4 (11/15)	0	13.3^c^ (2/15)	13.3^d^ (2/15)	93.3^e^ (14/15)
	**2**	27/01/17	Cull	60	100	0	0	0	8.3^f^ (5/60)
**2**		27/01/17	Report case	21	71.4 (15/21)	14.3 (3/21)	4.8^g^ (1/21)	9.5^h^ (2/21)	47.6^i^ (10/21)
		27/01/17	Carcasses	3	*Na*	*na*	*Na*	*na*	100
		30/01/17	Cull	40	100	0	0	0	0
**3**	**B**	27/01/17	Contact Tracing	60*	100	0	0	0	0
	**A**	30/01/17	Report case	20	100	0	0	0	0
	**A**	30/01/17	Carcasses	4	*Na*	*na*	*Na*	*na*	0
	**B**	30/01/17	Carcasses	3	*Na*	*na*	*Na*	*na*	66.7 (2/3)

Inc/IS, insufficient to test; *na*, not applicable.

Serology non-negative antibody titre 1/2–1/8; serology positive titre ≥1/16.

PCR % at flock level (positive Ct values <36, non-negative Ct 36–40, PCR negative ‘No Ct’).

* Enhanced sampling strategy for Case 3 contact tracing (60/60/60).

a H5N8 geomean titre = 4.9.

b H5N8 geomean titre = 32, overall 10.7 (60% population seroconverted, 12/20 birds).

c H5N8 geomean titre = 8.

d H5N8 geomean titre = 90.5 (26.7% population seroconverted, 4/15 birds).

e Only 6.7% (1/15 birds) PCR negative.

f 88% (53/60 birds) PCR negative.

g H5N8 geomean titre = 8.

h H5N8 geomean titre = 22.6, overall 16 (14.3% population seroconverted, 3/21 birds).

i Only 14.3% (3/21) birds PCR negative; 38.1% (8/21) birds had an inconclusive result.

### Serology

Sera were decanted from the clotted blood samples. All were screened by haemagglutination inhibition tests (HAIT) to detect virus subtype-specific antibodies against H5 and H7 AIV antigens, and APMV-1 antigens according to the internationally recognised standard methods [4, 12, 13]. The HAIT assays used a combination of H5 and H7 AIV standard antigens described in the annual AI poultry sero-surveillance programme in all EU Member States [Commission Decision 2010/367] to detect the presence of antibody to NAI (notifiable avian influenza) viruses which were A/teal/England/7394-2805/06 (H5N3), A/chicken/Scotland/59 (H5N1), A/turkey/England/647/77 (H7N7) and A/African starling/England/983/79 (H7N1). All sera were additionally screened with the H5 AIV antigen A/duck/England/036254/14 (H5N8).

### Virus isolation in embryonated SPF fowls' eggs

Cloacal and oropharyngeal swabs were each expressed into 1 ml of brain-heart infusion broth (BHIB) containing antibiotics. Liquid pools were then prepared by combining equal volumes of BHIB from groups of up to five swabs obtained from the same anatomical site [4]. The liquid swab pools and pooled diagnostic tissue homogenates (10% w/v clarified brain, trachea and lung, intestine and mixed viscera), collected from carcases were inoculated into 9- to 11-day-old specific pathogen free (SPF) embryonated fowls' eggs (EFE) according to internationally recognised standard methods for virus isolation [4, 13]. Allantoic fluids were harvested at 2 and 6 days post-inoculation and were tested by HAIT to confirm the presence of H5 virus, as described above.

### Nucleotide sequencing and phylogenetic analysis

In order to confirm the HPAIV status, H5 cleavage site (CS) sequencing was undertaken on selected clinical samples, using previously described methods [14]. CS sequences were derived from the following samples:

**Case 1**: pooled brain labelled as AB on a tree (Epi 1) and pooled intestines (Epi 2); **Case 2**: pooled brain and an OP swab; **Case 3**: C and OP swabs (A or B).

Egg-amplified virus (egg passage 1) was used for whole-genome sequencing, and for Case 1 was derived from pooled brain and pooled intestines (Epi groups 1 and 2 labelled as A&B on a tree) respectively), from the pooled brain for Case 2, and from an OP swab for Case 3. Viral RNA was extracted as described for RRT-PCR testing (without carrier RNA). Additionally, clinical material was assessed using conventional RT-PCR and Sanger sequencing with haemagglutinin (HA) gene-specific primers [14]. Primer sequences are available upon request. Nucleotide sequence alignments were performed with MAFFT version 7 [15]. The phylogenetic tree was inferred by Maximum Likelihood using IQ-TREE [16], with the best-fit nucleotide substitution model found using ModelFinder [17] and performing a phylogeny test of 1000 ultrafast bootstrap replicates [18]. The analysis involved 172 nucleotide sequences with 1716 nucleotide sites. The best-fit substitution model used was GTR + F + I + G4, and the tree with the highest log likelihood (−13537.2165) was selected.

### Statistical analysis

**Fig. 2.**
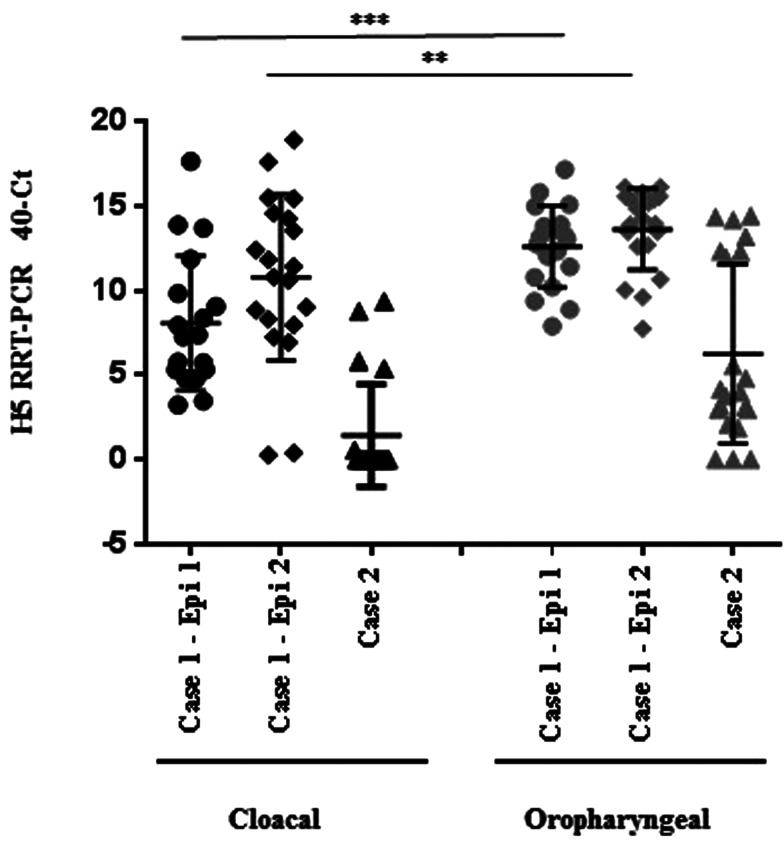
Comparison of molecular detection of viral RNA in cloacal and oropharyngeal swabs; H5 RRT-PCR 40-Ct values for pheasant cases 1, 2 and 3. Cloacal swabs shown in black, and oropharyngeal swabs shown in grey. Mean and standard deviation shown. **P < 0.01; ***P < 0.001.

Statistical significance between Ct values from the different molecular assays (M-gene, H5 and N8-specific RRT-PCR) were determined by ANOVA. Statistical significance between Ct values for OP swabs and C swabs (shown in Fig. 2) was determined by a two-tailed Student's *t* test, where *P* < 0.05 was deemed statistically significant.

## Results

### Molecular detection of HPAIV H5N8

Positive swabs for H5N8 viral RNA were only detected by RRT-PCR for Cases 1 and 2, using H5 and N8-specific RRT-PCRs and an M-gene screening assay. Interestingly, the M-gene assay was found to be more sensitive than the H5-specific assay (lower Ct values) for detecting viral RNA in pheasant samples (*P* = 0.03). All samples were negative for APMV-1 viral RNA.

**Fig. 3.**
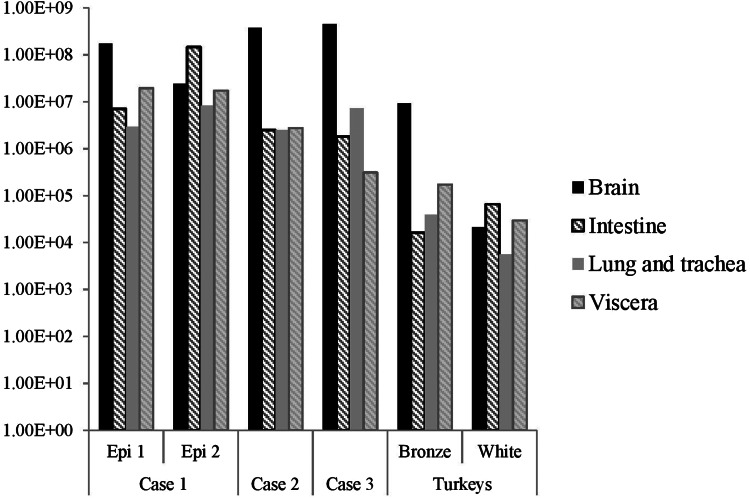
Distribution of viral RNA in tissues taken from submitted carcasses from pheasant Cases 1, 2 and 3, denoted by relative expression units (REU) through comparison with a series of A/chicken/Scotland/1959 H5N1 RNA standards, determined by H5-specific RRT-PCR.

The distribution of viral RNA in pooled organ samples taken from submitted carcasses was determined using an H5-specific RRT-PCR [6], and expressed as relative equivalent units (REU) [11, 19] through comparison with a series of A/chicken/Scotland/1959 H5N1 RNA standards (Fig. 3). In terms of tissue distribution, the tropism was similar to that observed in turkeys during the first case of the H5N8 2016–17 outbreak in GB, where viral RNA was widely distributed throughout the carcass, indicating systemic virus distribution typical of HPAIV infections with greater viral loads being detected in brain and intestine. A similar trend was observed using N8-specific RRT-PCR (data not shown).

Swab samples collected at cull were also subsequently submitted for Case 1 and Case 2; of these, 14/15 (93.3%) and 5/60 (8.3%) were positive for Case 1 Epi groups 1 and 2 respectively, whilst all cull swabs for Case 2 were negative (Table 2).

### Comparison of PCR detection in oropharyngeal and cloacal swabs

RNA extracted from OP swabs yielded lower Ct values compared to those from C swabs by all three molecular assays used (M-gene, H5 and N8), as shown for Case 1 and Case 2, reflecting the higher quantities of viral RNA in OP swabs as a result of preferential virus shedding by this route. There were no positive swabs detected for Case 3. The Ct differences observed for Cases 1 and 2 were approximately 3–4 (OP > C) across all three assays, with mean Ct differences by H5-specific RRT-PCR of 4.5, 2.8 and 1.3 for Case 1 Epi 1, Case 1 Epi 2 and Case 2 respectively. The lower Ct values obtained from OP swabs by H5 RRT-PCR is also highlighted in Figure 2, where data has been shown as ‘40-Ct value’ to provide a visual representation of positivity. For Case 1, these differences were statistically significant (Student's *t* test), where *P* < 0.001 and *P* = 0.01 for Epi groups 1 and 2 respectively. There was no significant difference when comparing OP and C swabs for Case 2 (*P* = 0.592), most likely due to the lack of Ct values obtained for the C swabs.

### Pathology and immunohistochemistry

Gross pathology findings in birds from all three cases were consistent with HPAIV infection in other gallinaceous species, although classical haemorrhagic lesions in lymphoid tissue were observed in only a small number of birds. However, a novel finding was the presence of diphtheroid plaques in the oropharyngeal mucosa, which were associated with the necrotising stomatitis induced by AIV infection of the epithelium.

**Fig. 4.**
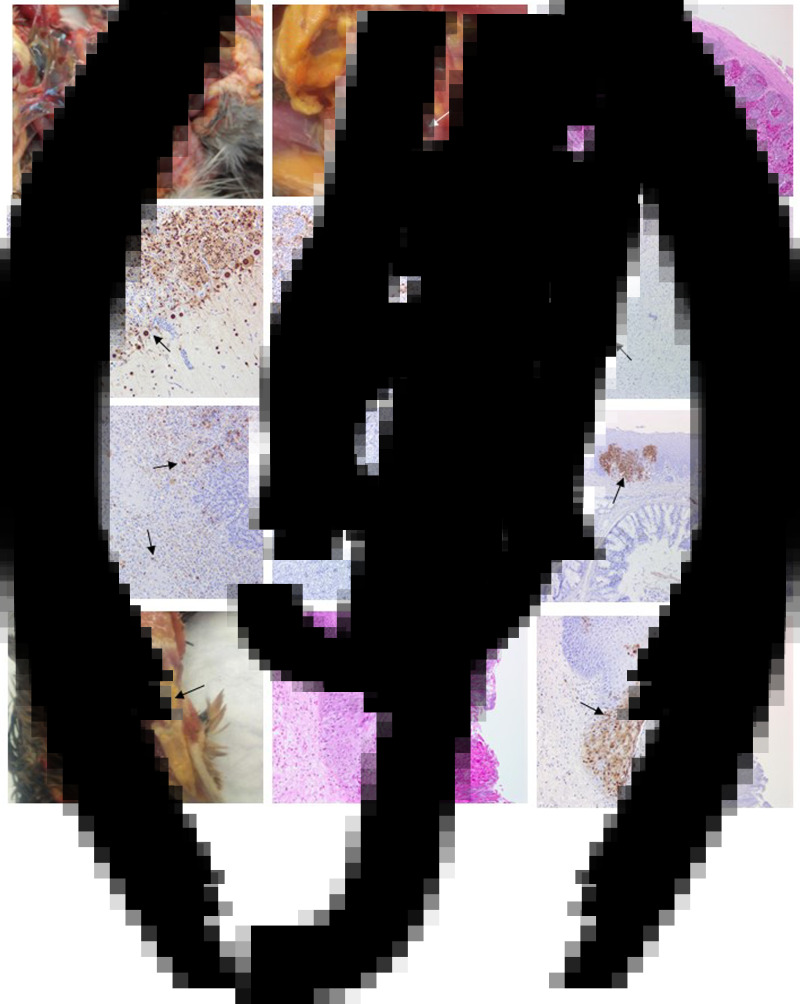
Pathological and immunohistochemical observations for Case 1 (a to i): haemorrhages in gut-associated lymphoid tissue (a); necrosis and haemorrhages in the pancreas (b); haemorrhages and lymphoid destruction in Peyer's patches in the jejunum (c); virus detection in the cerebellum (d), spleen (e), heart (f), pancreas (g), kidney (h) and oral mucosa (i). Pathological and immunohistochemical observations for Case 2 (j to l): Diphtheroid plaques in oral mucosa (j); necrotising stomatitis (k); demonstration of intralesional virus antigen (l).

Four birds from Case 1 were originally submitted, two pheasants from each Epi group, and gross pathological findings at post mortem in both Epi groups were similar. One of the birds from each group presented with no significant lesions while the other bird from each Epi group displayed multifocal haemorrhages of the mucosa-associated lymphoid tissue of the intestinal tract (Fig. 4a) and moderate splenomegaly and pulmonary hyperaemia. There were also smaller petechiae in the serosal membranes, haemorrhages in the proventriculus (smaller, multifocal petechial rather than the classic glandular haemorrhage); both had splenomegaly and heavy hyperaemic lungs, whilst a bird from Epi group 1 also had white foci and petechiae in the pancreas (Fig. 4b). Four additional pheasants from Case 1, two from each Epi group, were submitted 2 days later and gross examinations and sampling were also conducted. One bird from each Epi group was unremarkable, whereas the other one showed pancreatic mottling (necrosis) and haemorrhages (Fig. 4b) and splenomegaly. No lesions in lymphoid tissue were observed. Gross observations at post mortem were supported by histopathology (Supplementary Table S1 – columns A and B). For all eight birds examined from Case 1, necrosis and haemorrhages of gut-associated lymphoid tissue (GALT) were observed in three of the birds. Splenic necrosis (*n* = 3/8), necrotising pancreatitis (*n* = 3/8) and rhinitis (5/8) were also observed. Immunohistochemical distribution of virus antigen for Case 1 is summarised in Supplementary Table S2 (columns A and B). There was systemic virus replication, with marked lymphotropism and endotheliotropism in the two birds displaying GALT haemorrhages and necrosis, less prominent in others (Supplementary Table S2 – columns A and B). Virus antigen was detected consistently in parenchymal and endothelial cells in encephalon, heart and nasal mucosa, and less frequently in kidney, pancreas and liver (Figs 4c–4i). Interestingly, replication in the oropharyngeal epithelium was frequently observed.

For Case 2, three pheasants underwent pathological investigation at post mortem, and observations were similar to Case 1, although serosal and lymphoid haemorrhages were not seen. Gross pathology included pancreatic necrosis and haemorrhages (1/3 birds) and splenomegaly (1/3 birds). All three birds also displayed white to yellow diphtheric plaques in the oropharyngeal mucosa (including, palate, tongue and glottis) (Fig. 4j). Histopathological changes (Supplementary Table S1 – column C) and virus distribution (Supplementary Table S2 column C) were similar to those described for Case 1 with the addition of a severe necrotising and ulcerative stomatitis with intra-lesional viral antigen and secondary bacterial infection (Figs 4k, 4l). There was marked replication in oropharyngeal mucosa and salivary glands. Additionally, virus particles were detected in tissues from Case 2 by transmission electron microscopy (TEM) (data not shown).

For Case 3, OP and C swabs were taken from the seven submitted carcasses (four from Epi group A, three from Epi group B), and only two birds from Epi group B were positive by RT-PCR. These two birds underwent pathological investigation, and gross observations included diphtheroid plaques in the oropharyngeal mucosa and splenomegaly (2/2 birds), and pancreatic necrosis and haemorrhages (1/2 birds). Histopathological changes (Supplementary Table S1 – column D) and viral antigen distribution (Supplementary Table S2 – column D) were similar to Cases 1 and 2.

### Serology

Serology results for all cases are summarised within Table 2. For Case 1, Epi group 1 had positive antibody titres against subtype/clade matched antigen (H5N8-2014) in 25% (5/20) of serum samples (titres ≥1/16; geomean titre 32), and a further 35% (7/20) of sera with weak/non-negative titres (1/2–1/8; geomean titre 4.9). In summary, 12/20 (60%) of birds sampled from Epi group 1 during initial disease investigation had seroconverted, and the overall geomean titre for this Epi group was 10.7. The ‘cull’ serology within Epi group 1 also detected positive titres (≥1/16) in 13.3% of serum samples (geomean titre 90.5), together with weak/non-negative titres (1/2–1/8) in 13.3% of samples (geomean titres 8). Overall, 4/15 birds culled within Epi group 1 had detectable antibody titres, suggesting that 26.7% of the population had seroconverted. In comparison, Epi group 2 serology (disease suspicion and cull) were 100% negative suggesting a difference in the timeframe of exposure for the two Epi groups.

Positive antibody titres were also detected during the initial disease investigation for Case 2. These were most likely due to previous exposure to the virus, and almost certainly induced following the transfer of pheasants from Case 1. Positive titres were detected in 9.5% (2/21) of serum samples (≥1/16; geomean titre 22.6), and weak/non-negative titres detected in 4.8% (1/21) of samples (1/2–1/8; geomean titre 8). Overall, 3/21 (14.3%) birds sampled in Case 2 had seroconverted, with an overall geomean titre of 16. In comparison, all ‘cull’ serology for Case 2 was negative. All serology for Case 3 was also negative. Some birds from Cases 1 and 2 demonstrated reactivity against APMV-1 antigens, although this was not vaccine-associated reactivity as all vaccines are prohibited for gamebirds in the United Kingdom. Given the obvious sub-clinical and recovered effects in some individual birds, we propose that these responses were naturally induced as a result of non-fatal infection.

### Virus isolation

Samples that were positive for virus isolation were derived from pooled brain and pooled intestines (Case 1, Epi groups 1 and 2 respectively), pooled brain and pooled OP swabs (Case 2) and an OP swab (Case 3, Epi group B). For Case 3, virus isolation was not undertaken for Epi group A due to negative molecular and serology results. For all three cases, haemagglutinating agents (HA) were detected following passage in EFE, with HA titres ranging between 1/2 and 1/128. Conventional HAIT typing with a live virus isolate confirmed H5N8 virus subtype for Cases 1 and 2 (not undertaken for Case 3).

### Sequence analysis

For all three cases, the HPAIV status was confirmed by H5 CS sequence, directly from clinical specimens. The CS motif was identified as PLREKRRKRGLF, which was identical to that found in other 2016–17 European viruses including other GB poultry cases.

**Fig. 5.**
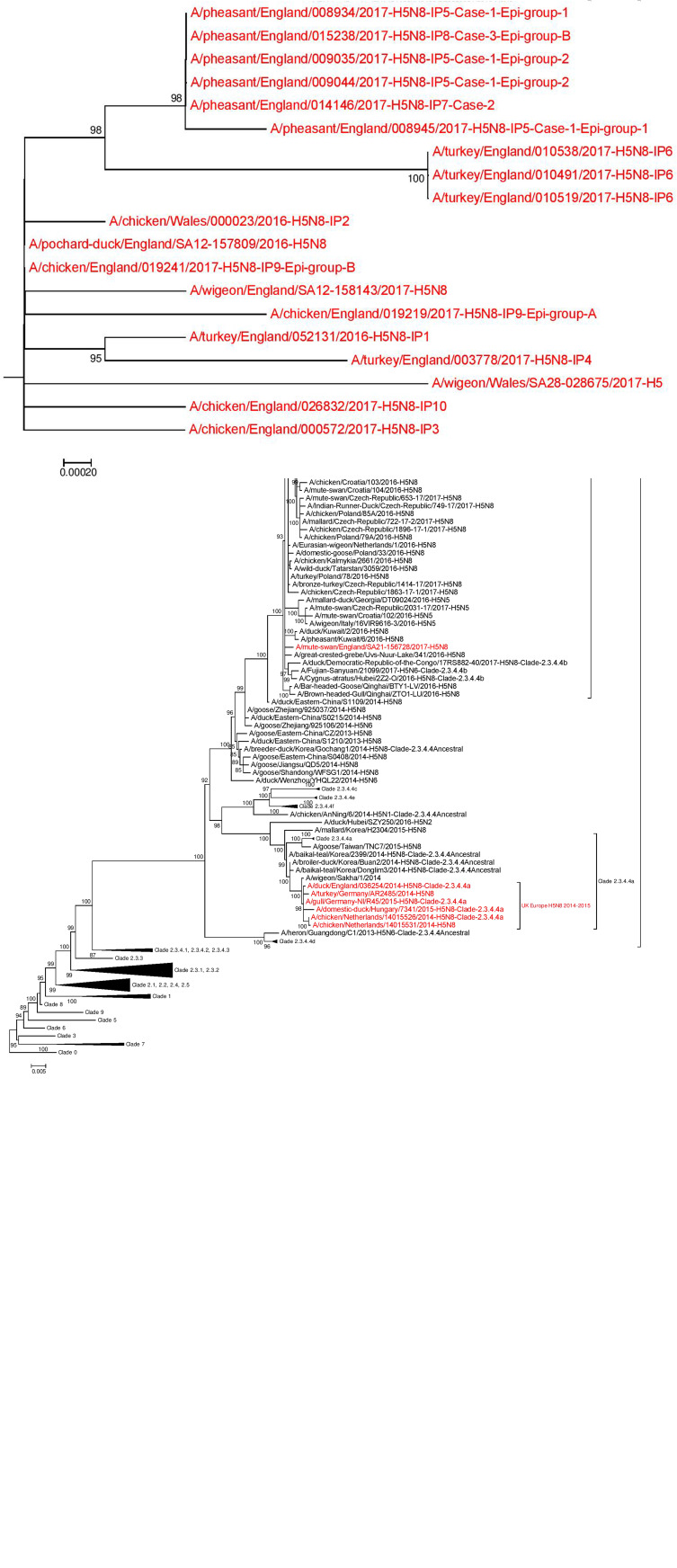
(a): Phylogenetic tree inferred by Maximum Likelihood using IQ-TREE based on 172 nucleotide sequences with 1716 nucleotide sites. Best-fit nucleotide substitution model determined using ModelFinder and performing a phylogeny test of 1000 ultrafast bootstrap replicates. Tree based on the HA gene for poultry and wild bird H5N8 cases, with an amplified section of the HA gene phylogenetic tree highlighting the pheasant cluster (inset box – b); UK H5N8 sequences, including the pheasant cluster, highlighted red.

Phylogenetic analysis of the HA gene placed the sequences derived from the ‘pheasant cluster’ (Cases 1, 2 and 3; IP 5, 7, 8) within the north/western European clade 2.3.4.4b (Fig. 5). Accession numbers on GISAID of the HA sequences were as follows: A/pheasant/England/008934/2017 – EP1922172 (from CS sequencing); A/pheasant/England/015238/2017 – EP1922326 (from complete genome sequencing); A/pheasant/England/009035/2017 – EP1922310 (from complete genome sequencing); A/pheasant/England/009044/2017 – EP1922173 (from CS sequencing); A/pheasant/England/014146/2017 – EP1922318 (from complete genome sequencing) and A/pheasant/England/008945/2017 – EP1922290 (from complete genome sequencing) The clustering of the three viruses from these pheasant properties placed them as highly similar but distinct from other GB poultry viruses and also differentiated them from wild bird strains but again with extremely high similarity including up to 99.9% with a ‘late 2016’ wild bird strain (A/pochard/England/157809/2016) (Fig. 5a). Across the whole genome (eight segments), the sequences from all three pheasant cases were very similar, with at least 99.95% (99.95–99.99%) nucleotide identity with each other. There were two coding nucleotide changes identified in the HA gene, which translated to D503N for Case 1 Epi group 1 (sequence 8945), and N236N/H for Case 2 (sequence 14146); both sequences derived from whole-genome sequencing of egg pools (inoculated with pooled intestines and a swab respectively). However, the mutation in sequence 8945 was not reflected in additional HA sequences generated by conventional Sanger sequencing from clinical material for both Epi groups in Case 1; sequence 8934 for Epi group 1 (derived from pooled brain), and sequence 9044 for Epi group2 (derived from pooled intestines). This suggests that these HA mutations may have been acquired during passage in EFE. Additionally, when comparing whole-genome sequence for all three pheasant cases (egg pool derived), there was only one further coding nucleotide change identified, which translated to E388D in the neuraminidase (NA) protein for Case 1 Epi group 1, again potentially acquired during the single egg passage. All other genes were 100% identical at the nucleotide level, reflecting the close genetic relationship between the pheasant virus sequences derived from the reported outbreak.

### Summary of results

The combined results (molecular and serological) or all three pheasant cases, together with results from subsequent cull/research samples, are summarised in Table 2. The results and timeline suggest that a wild bird exposure/incursion led to the infection of pheasants in Case 1. When considered together, the molecular and serological results for Case 1 suggest that there was a difference in the timeframe of exposure for the two Epi groups, with Epi Group 1 more likely to have been infected before Epi Group 2. At the time of sampling, Epi group 1 appeared to have been infected earlier and birds were starting to recover (positive PCR and serology results), whilst Epi Group 2 was still actively infected (positive PCR results only and no seroconversion). As part of the pheasant rearing process, infected birds were transferred from Case 1 property to the sites involved in Case 2 and Case 3 prior to the detection of clinical disease; the movement of birds between the sites is summarised in a schematic (Fig. 1).

## Discussion

Outbreaks of HPAIV have a significant impact on the poultry industry; along with negative publicity, there is a significant economic impact for the farmer, together with consequences for trade at both the national and international level. An outbreak on a gamebird premises can also cause a significant financial and trading impact for the farmer and related leisure industries. In this study, we report on disease presentation and epidemiology following natural infection in pheasants with HPAIV H5N8 (clade 2.3.4.4b) during a small epizootic in England in January 2017. Infected birds exhibited a range of clinical signs, from inapparent to mild, moderate and severe, culminating in rapid mortality for a limited proportion. However, it should be noted that the disease may have been exacerbated by an underlying *Mycoplasma* infection. The clinical presentation for H5N8 HPAIV in pheasants was not classical, but more similar to that reported for ND in game birds [20], typified by a relatively long lag phase with an extended clinical course following earlier disease suspicion. However, even pheasants exhibiting mild clinical signs still maintained significant levels of virus replication and shedding, and the detection of viral RNA in a wide range of tissues indicated that the virus has the potential to disseminate systemically within the host. Gross pathological observations were consistent with HPAIV, although only a small number of examined birds had classical haemorrhagic lesions in lymphoid tissue. However, the presence of diphtheroid plaques in the oropharyngeal mucosa associated with necrotising stomatitis induced by AIV infection of the epithelium was a novel but consistent finding in these cases of the disease. This observation may be useful for disease recognition in the field, although other differential diagnoses including poxvirus infection and vitamin deficiencies would need to be excluded as part of differential diagnosis. The clinical observations and tissue distribution observed in pheasant cases during the 2016–17 H5N8 HPAIV outbreak in GB appear consistent with pheasants infected with other HPAIV serotypes. Previous studies have shown that a flock of pheasants naturally infected with H5N2 HPAI where demonstrated 10% mortality overnight, whilst surviving pheasants exhibited only mild clinical signs [21]. Despite mild clinical signs, viral antigen was detected in a wide range of organs, with the consistent gross pathological finding of congestion of cerebellar meningeal blood vessels. Furthermore, the histopathological findings and virus distribution by IHC are similar to pheasants experimentally infected with A/Chicken/Hong Kong/220/97 H5N1 HPAIV [22]. Similarly, experimental intranasal, intraocular or intra-tracheal infection of pheasants with high dose10^7^ ELD_50_ (50% egg lethal dose) HPAIV H5N3 did not induce any detectable clinical signs within a 14-day observation period, although virus shedding was detected up to 14 days post-inoculation [23]. In addition to reports of H5Nx HPAIV in pheasants in the United States since the 2016–17 H5N8 HPAIV outbreak in GB [24, 25], there have been cases of H5N8 HPAIV infection in pheasants elsewhere in Europe, but similarly these have been typified by a lack of clinical indicators in most cases. However, cases in Germany and the Czech Republic have been associated with individual birds or small backyard flocks of mixed species, rather than the larger flock sizes found to be infected in England (International Reference Laboratory for AI, APHA-Weybridge – Personal communication). In Bulgaria, pheasant infections were also detected in mixed farms in 2017, with limited clinical signs observed (weakness, conjunctivitis) and low mortality.

In this study, there were subtle variations in presentation amongst the cases. The very low mortality and disease signs observed for Case 3 suggest that the virus infection may have been restricted to a small number of pens on this site and had not spread widely. This is supported by previous studies, where relatively low levels of virus were detected in environmental samples following experimental infection of chickens and ducks with H5N1 HPAIV, despite significant levels of virus shedding in both species [26]. Furthermore, nine additional tracing sites potentially receiving birds from infected pheasant properties were identified during surveillance, and all pheasants sampled (swabs and blood) from these sites were diagnosed as negative for H5 HPAIV, suggesting that the virus did not spread or transmit under these circumstances. Genetic analysis of England H5N8 HPAIV revealed that the genotype was as previously reported in other contemporary European outbreaks [2], and suggests the three infected pheasant properties shared a close causal association. The results suggest that H5N8 HPAIV was most likely introduced onto one of these properties via wild birds (precise primary premise uncertain as the level of resolution is low) with subsequent spread to the other two sites. However, during this process of spread and transmission, the viruses showed negligible genetic change although the time period from disease suspicion to final depopulation and sampling was only 2 weeks.

The introduction and spread of H5N8 HPAIV within the England ‘pheasant cluster’ had in part been exacerbated by the processes involved in the rearing of pheasants, which includes the frequent movement of birds. The serology data indicated differences in the timeframe of exposure for each of the cases, and supported the timeline for the movement of birds between sites. Firstly, the birds associated with Case 1 were over-wintering in fields, kept in ‘enclosures’ that were open to the environment and easily accessible to wild birds. This site was situated adjacent to the sea, with Epi group 2 bordered by a sea wall, and numerous areas of open water in the vicinity. The known large populations of wild birds, especially migratory waterfowl, in the surrounding area were therefore the most likely source of infection for Case 1, as these wild birds were observed on the pheasant properties in the vicinity of the pheasants' food. Indeed, there were two detections of H5N8 HPAIV in wild birds (a wigeon and a greylag goose), located within 80 km of the pheasant cluster. The differing morbidity between the two Epi groups in Case 1 may have been due in part to differences in the timeframe of exposure (the results suggest that Epi group 1 pre-dated Epi group 2). In comparison, the pheasants in Cases 2 and 3 were caged, limiting contact between birds on these sites, and limited opportunity for contact with wild birds. This suggests that the underlying source of infection for Cases 2 and 3 was most likely associated with the movement of pheasants between sites, where birds from Case 1 (Epi group 2) were transferred to another location for vaccination (Case 3), before being sent on to the rearing property (Case 2) where males and females were housed together for breeding purposes. Indeed, the nature of the gamebird rearing process requires frequent movement of birds, which constitutes the single biggest risk factor for the spread of infection, and makes it difficult to control lateral spread between premises. In particular, the movement of sub-clinically infected birds may pose a biosecurity risk. Additionally, husbandry practices had limited hygiene considerations, therefore any movement of personnel or equipment between sites could have also increased the risk of fomite spread of virus between sites. However, despite the increased risk of virus spread between sites, the caged housing systems used at the premises associated with Cases 2 and 3, along with the reduced pheasant susceptibility to virus, would have limited the opportunity for virus spread once within the site. The disease could still have spread within each pen that had received an infected bird, although it was difficult to epidemiologically sample the populations on these sites, as the penning system effectively divided the birds into a large number of sub-populations. In order to mitigate this, an enhanced sampling strategy was implemented for the contact tracings in breeding units of ten birds (including Case 3, Epi group B), where three out of ten birds from twenty pens were sampled. Additionally, there should also be some caveats around the lack of seroconversion in Case 3, since a lack of seroconversion at pen level may not be fully reflective of the infection status at this level. There is the potential for birds that test seronegative to be in the same pen with birds that are seropositive, particularly if the seropositive birds were not infectious when placed, or there was a lack of efficient transmission, even though the contact opportunity was high. Furthermore, additional contact sites did not indicate further onward transmission of the virus despite the movement of pheasants between sites. This suggests that the risk of infection was most likely dependent upon the timing and level of exposure to infected pheasants or fomites, with the possibility that the 2016–17 clade 2.3.4.4.b strain of H5N8 may not have the propensity to transmit and become fully established in pheasant populations. Further experimental studies would be needed to confirm this hypothesis, as it may have important implications for surveillance, where clinical parameters may not be a reliable indicator of infection in pheasants located in ‘at-risk’ locations.

The processes involved in gamebird rearing also led to difficulties in robustly sampling the affected premises, where the penning systems used, along with the movement of birds, could have led to uncertainties. Therefore, an enhanced sampling strategy was implemented for all the contact traced pheasant breeding sites, to take into account the subdivision of a former epi group of birds. It is recommended that the same sampling approach is applied to future suspected cases of AI in gamebirds. Additionally, veterinary examinations need to be detailed, and undertaken by veterinarians with sufficient expertise in poultry and gamebirds, in order to ensure that clinical presentation is efficiently detected. Furthermore, the significant differences observed in PCR test results obtained for oropharyngeal and cloacal swabbing, suggestive of preferential virus shedding via the oropharyngeal route, was similar to that observed for other Galliforme species with respect to this virus strain (APHA unpublished data), and may provide useful evidence for streamlining the sampling protocol on suspicion of disease in pheasants. In conclusion, the data presented here does not support that H5N8 HPAIV infection was easily established in farmed pheasants, although a different clinical picture may potentially be observed under different circumstances and within different housing systems and at different stages of production.

## Data Availability

The datasets used and/or analysed for the completion of this manuscript are available from the corresponding author on reasonable request.

## Supplementary material

For supplementary material accompanying this paper visit http://doi.org/10.1017/S0950268821002740. click here to view supplementary material
