# Supplementary material for: Incursion of H5N8 high pathogenicity avian influenza virus (HPAIV) into gamebirds in England
Source: Epidemiol Infect. 2022 Feb 10;150:e51. doi: 10.1017/S0950268821002740 (PMC8915197; doi:10.1017/S0950268821002740)
Supplement: Supplementary file 1 [file hygsup.zip › S0950268821002740sup001.doc]

**Supplementary Table S2:** Immunohistochemical detection of Influenza A nucleoprotein in pheasants

|  | **A** | | | | | | **B** | | | | | **C** | | | **D** | |
| --- | --- | --- | --- | --- | --- | --- | --- | --- | --- | --- | --- | --- | --- | --- | --- | --- |
| **Case** | **1** |  |  |  | |  | | **1** |  |  |  | **2** |  |  | **3** |  |
| Epidemiological Unit | 1 | 1 | 2 | 2 |  | | 1 | | 1 | 2 | 2 | - | - | - | 2 | 2 |
| Bird Number | 1 | 2 | 3 | 4 |  | | 1 | | 2 | 3 | 4 | 1 | 2 | 3 | 1 | 2 |
|  |  |  |  |  |  | |  | |  |  |  |  |  |  |  |  |
| Skin | - | N/a | - | - |  | | - | | +/- | - | - | - | - | - | - | N/a |
| Muscle | - | N/a | +/- | - |  | | - | | - | - | - | - | - | - | - | N/a |
| Brain | +++ | ++ | ++ | +++ |  | | +++ | | + | ++ | ++ | ++ | +++ | +++ | ++ | + |
| Heart | ++ | ++ | ++ | +/- |  | | +/- | | + | +/++ | +/++ | +/++ | +/++ | + | +/++ | + |
| Spleen | +/- | ++/+++ | ++ | - |  | | - | | ++ | - | + | +/- | - | N/a | + | +/- |
| Liver | + | ++ | ++ | - |  | | +/- | | + | +/- | ++ | +/- | - | N/a | - | - |
| Kidney | + | ++ | ++ | - |  | | - | | +/- | - | +/- | +/- |  | + | + | +/- |
| **Adrenal gland** | ++ | N/a | N/a | N/a |  | | - | | N/a | N/a | + | N/a | + | N/a | ++ | + |
| Pancreas | ++ | + | - | - |  | | - | | - | ++ | ++ | ++ | ++ | N/a | +/++ | - |
| Ovary / Testes | O +/- | O ++ | O ++ | O - |  | | N/a | | O + | O - | O- | O- | O - | O - | O - | O +/- |
| Nasal cavity | + | ++ | ++ | +/- |  | | - | | + | +/- | +/++ | ++ | +/- | +/- | ++ | ++ |
| Trachea / Bronchi | - | + | + | - |  | | - | | +/- | +* | - | - | - | - | - | - |
| Lung | +/- | ++ | + | - |  | | - | | + | N/a | - | - | - | N/a | - | +/- |
| Air Sacs | - | N/a | + | - |  | | N/a | | +/- | - | N/a | + | N/a | N/a | + | N/a |
| Oropharynx | + | ++ | ++ | - |  | | -// + | | +/-//++ | -// + | +/-*//+ | ++ | ++ | ++ | ++ | ++ |
| Gizzard | - | ++ | + | - |  | | N/a | | + | + | N/a | - | - | N/a | + | + |
| Proventriculus | - | ++ | ++ | - |  | | N/a | | + | - | N/a | - | - | N/a | - | - |
| **Duodenum** | + | ++ | + | - |  | | - | | - | - | - | - | - | N/a | - | - |
| Caecal Tonsil | +/- | ++ | ++ | - |  | | - | | +/++ | +/- | N/a | - | - | N/a | - | +/-* |
| Ileum | +/- | ++ | + | +/- |  | | - | | +/++ | +/- | N/a | - | - | N/a | - | - |
| Jejunum | +/- | ++ | + | - |  | | - | | + | - | +/-* | - | - | N/a | +* | - |
| Colon | +/- | + | + | - |  | | +/- | | + | +/- | N/a | +/-* | - | N/a | +/-* | - |
| **Endothelium overall** | **+/-** | **++** | **+/++** | **-** |  | | **-** | | **+** | **-** | **+/-** | **+/-** | **-** | **+/-** | **+/-** | **-** |
