# Supplementary material for: Incursion of H5N8 high pathogenicity avian influenza virus (HPAIV) into gamebirds in England
Source: Epidemiol Infect. 2022 Feb 10;150:e51. doi: 10.1017/S0950268821002740 (PMC8915197; doi:10.1017/S0950268821002740)
Supplement: Supplementary file 1 [file hygsup.zip › S0950268821002740sup002.doc]

**Supplementary Table S1:** Histopathology of AIV in pheasants

|  | **A** | | | | | **B** | | | | | **C** | | | | **D** | |
| --- | --- | --- | --- | --- | --- | --- | --- | --- | --- | --- | --- | --- | --- | --- | --- | --- |
| **Case** | **1** |  |  |  |  | **1** |  |  |  |  | **2** |  |  |  | **3** |  |
| Epidemiological Unit | 1 | 1 | 2 | 2 |  | 1 | 1 | 2 | 2 |  | - | - | - |  | 2 | 2 |
| Bird Number | 1 | 2 | 3 | 4 |  | 1 | 2 | 3 | 4 |  | 1 | 2 | 3 |  | 1 | 2 |
| Brain |  |  |  |  |  |  |  |  |  |  |  |  |  |  |  |  |
| Non suppurative encephalitis | + | + | + | + |  | + | - | +/++ | - |  | + | + | ++ |  | +/++ | ++ |
| Heart |  |  |  |  |  |  |  |  |  |  |  |  |  |  |  |  |
| Necrotizing myocarditis | + | - | - | - |  | - | - | + | +/- |  | - | +/++ | - |  | +/- | - |
| Spleen |  |  |  |  |  |  |  |  |  |  |  |  |  |  |  |  |
| Necrosis |  | ++ | + |  |  | N/a | ++ |  |  |  |  |  |  |  | ++ | +/- |
| Liver |  |  |  |  |  |  |  |  |  |  |  |  |  |  |  |  |
| Necrosis | +/- | - |  |  |  | - | - | - | + |  |  |  | - |  |  |  |
| Kidney |  |  |  |  |  |  |  |  |  |  |  |  |  |  |  |  |
| Tubular necrosis | +/- | - | - |  |  | - | - | - | - |  | - | - |  |  | - | - |
| **Adrenal** |  |  |  |  |  |  |  |  |  |  |  |  |  |  |  |  |
| Necrosis | ++ | N/a | N/a |  |  | - | N/a | N/a | - |  | - | +/++ | N/a |  | + |  |
| Pancreas |  |  |  |  |  |  |  |  |  |  |  |  |  |  |  |  |
| Necrotizing pancreatitis | ++ | - | - | - |  | - | - | ++ | ++ |  | ++ | ++ | N/a |  | ++ |  |
| Nasal cavity |  |  |  |  |  |  |  |  |  |  |  |  |  |  |  |  |
| Lymphoplasmacytic rhinitis | + | - | - | +/- |  | - | + | + a | +/++* |  | + | - |  |  |  | +/- |
| Air Sacs |  |  |  |  |  |  |  |  |  |  |  |  |  |  |  |  |
| Necrotizing air sacculitis | ++ | N/a | +/- |  |  | N/a | N/a | - | N/a |  | +/- |  | N/a |  |  | N/a |
| Oropharynx |  |  |  |  |  |  |  |  |  |  |  |  |  |  |  |  |
| Necrotizing stomatitis |  |  |  |  |  |  |  | + | + |  | ++ | +++ | +++ |  | +++ | +++ |
| Cecal Tonsil |  |  |  |  |  |  |  |  |  |  |  |  |  |  |  |  |
| MALT Haemorrhages |  | + | ++ |  |  | - | - | - |  |  |  |  | N/a |  |  |  |
| MALT necrosis |  | + | +/- |  |  | - | +/- | - |  |  |  |  |  |  |  |  |
| Ileum |  |  |  |  |  |  |  |  |  |  |  |  |  |  |  |  |
| MALT Haemorrhages |  | + | - |  |  | - | +/- | - |  |  |  |  | N/a |  |  |  |
| MALT necrosis |  | + | - |  |  | - | - | - |  |  |  |  |  |  |  |  |
| **Duodenum** |  |  |  |  |  |  |  |  |  |  |  |  |  |  |  |  |
| MALT necrosis |  | + |  |  |  | - | +/- |  |  |  |  |  | N/a |  |  |  |
| MALT Haemorrhages |  | + | +/- |  |  |  |  |  |  |  |  |  |  |  |  |  |
| Jejunum |  |  |  |  |  |  |  |  |  |  |  |  |  |  |  |  |
| MALT necrosis |  | + | ++ |  |  |  |  |  |  |  |  |  | N/a |  |  |  |
| MALT Haemorrhages |  | + | +++ |  |  |  |  |  |  |  |  |  |  |  |  |  |
